# Preparation of Rumex abyssinicus based biosorbent for the removal of methyl orange from aqueous solution

**DOI:** 10.1016/j.heliyon.2023.e22447

**Published:** 2023-11-20

**Authors:** Mikiyas Abewaa, Eba Adino, Ashagrie Mengistu

**Affiliations:** a Department of Chemical Engineering, College of Engineering and Technology, Wachemo University, P. O. Box 667, Hossana, Ethiopia; b Department of Chemical Engineering, School of Mechanical, Chemical and Materials Engineering, Adama Science and Technology University, P.O. Box 1888, Adama, Ethiopia; c The Federal Democratic Republic of Ethiopia, Manufacturing Industry Development Institute, P. O. BOX 1180, Addis Ababa, Ethiopia

**Keywords:** Biosorbent, Environmental pollution, Methyl orange, Rumex abyssinicus, Wastewater treatment

## Abstract

Methyl orange is abundantly present in wastewater generated from textile industries causing serious human health and environmental problems. Therefore, this study was aimed at preparing a low cost and effective biosorbent from the stem of Rumex abyssinicus plant for the removal of methyl orange from aqueous solution. Characterization of the prepared adsorbent material was carried out using a pH point of zero charge, Scanning Electron microscope (SEM), Fourier Transform Infrared (FTIR) spectroscopy and X-Ray Diffraction (XRD). The design and optimization of methyl orange batch adsorption was carried out using the Box Behnken approach to Response Surface methodology aiming to reduce the experimental runs and time with adequate results. The characterization of the adsorbent revealed 7.9 (pHpzc), porous and heterogonous surface (SEM), presence of multiple functional groups (FTIR) and amorphous structure (XRD). The maximum removal efficiency of 98.5 % was found at pH, contact time, Methyl orange concentration and adsorbent dosage of 6, 60 min, 20 mg/L and 0.2 g/100 mL respectively. The isotherm studies were carried out using Langmuir, Freundlich, Toth and Koble Corrigan models in which Freundlich isotherm with a maximum R^2^ of 0.95 was found to fit data best showing heterogeneous and multilayer surface interaction. On the other hand, a kinetics study revealed that pseudo-second-order fitted the data best. Moreover, the thermodynamics analysis showed the nature of the adsorption to be endothermic, spontaneous and feasible. Generally, this work proved that the low-cost, environmentally friendly and easily prepared Rumex abyssinicus-based material could be an alternative adsorbent for dye detoxification at an industrial scale.

## 1 Introduction

Industrialization and urbanization have brought significant advancements and economic growth to societies worldwide. However, along with these benefits, they have also brought about various environmental challenges. One of the critical issues associated with industrialization is the impact on water resources. These processes have had profound impacts on water resources, leading to increased water demand and water pollution, particularly through the discharge of industrial wastewater [[1], [2], [3]]. According to studies [[4], [5], [6]], the rapid growth of industries and urban areas has resulted in the depletion and deterioration of surface and ground water quality, posing significant challenges to achieving sustainable development goals. The discharge of untreated or inadequately treated industrial wastewater into water bodies has led to the contamination of aquatic ecosystems, posing risks to public health and environmental sustainability [7,8]. The pollutants present in industrial wastewater can include heavy metals, organic compounds, and other toxic substances. These pollutants can disrupt the ecological balance of aquatic environments, harm aquatic organisms, and ultimately affect the quality of water available for human consumption and other purposes. As water scarcity becomes a pressing issue, it is crucial to address the detrimental effects of water pollution and develop effective strategies for wastewater treatment and management in industrial sectors [9,10].

One of the common pollutants found in wastewater from different manufacturing is dyes. Dyes are widely used in various industries, including textile, pharmaceutical, food, and cosmetic, due to their vibrant colors and dyeing properties [11,12]. Different types of dyes, such as reactive, direct, acid, and basic dyes, are employed based on their chemical properties and application requirements [13,14]. Among the commonly used dyes, methyl orange stands out as a representative azo dye with distinct properties and applications. Methyl orange dye, with the chemical formula C14H14N3NaO3S, is a synthetic compound widely used in various industries. It appears as a reddish-orange powder and is soluble in water. Methyl orange is known for its bright orange color and is commonly used as an acid-base indicator in laboratories and as a dye in textile and leather industries [15,16]. However, the extensive use of methyl orange has raised concerns about its environmental and human health impacts. Methyl orange is known to be toxic to aquatic organisms and can cause skin and eye irritation in humans [17,18].

Several treatment methods have been developed to address the decolorization of methyl orange-containing wastewater or aqueous solutions. Conventional treatment methods used for the removal of methyl orange dye from wastewater face several limitations. These limitations include incomplete removal, where traces of residual dye remain in the treated water, leading to environmental concerns [19]. Additionally, high chemical usage increases operational costs and generates chemical sludge. Moreover, pH sensitivity poses a challenge, as optimal pH conditions are required for effective dye removal. Recently, advanced wastewater treatment technologies have shown promise in effectively reducing the concentration of methyl orange and other pollutants [14,20,21]. These technologies include processes such as photocatalysis, electrochemical oxidation, membrane filtration, and biological treatment. Each of these methods has its merits and limitations. For instance, photocatalysis can efficiently degrade methyl orange molecules under suitable conditions but may require expensive catalysts and prolonged treatment times [15,22]. Electrochemical oxidation offers a rapid and effective approach but can be energy-intensive. Membrane filtration techniques can achieve high removal efficiency but may be limited by fouling issues [23,24]. Biological treatment methods can be cost-effective and environmentally friendly, but they may require longer treatment durations and specific operating conditions. These technologies offer advantages such as complete dye degradation and efficient removal of dye molecules. However, they also have limitations, including high energy requirements, generation of potentially harmful byproducts, and fouling issues [25,26].

Adsorption is a commonly employed technique for the removal of dyes from wastewater. Various types of adsorbents have been investigated for their effectiveness in the detoxification of pollutants, such as methyl orange dye. Among the commonly used adsorbents, commercial activated carbons (CACs) have shown remarkable adsorption abilities due to their extensive surface area [[27], [28], [29]]. However, the high cost associated with CACs has led to the exploration of alternative adsorbents, such as biomass-based activated carbons (BACs). BACs have gained attention as they offer a cost-effective solution for wastewater treatment [30,31]. The production of BACs involves tedious steps, including carbonization and activation processes, which enhance their adsorption capacity. These biomass-based adsorbents have demonstrated promising results in the removal of methyl orange dye from wastewater, highlighting their potential for sustainable and efficient detoxification methods [32,33]. Furthermore, the cost analysis of BACs compared to CACs reveals the significant cost savings associated with the use of biomass-based materials, making them a viable alternative for large-scale applications [32]. Contrary to this, the tedious preparation steps followed, the large amount of chemical consumed and activation at an elevated temperature made the biomass based activated carbon uneconomical and time consuming. Hence, searching for the potential biomass that does not need the activation or requires minor treatments, while demonstrating comparable adsorption capability, has become the target of researchers. In this regard, untreated powdered Rumex abyssinicus stem based biosorbent has been selected as cost effective and sustainable material for detoxification of persistent pollutants like MO from aqueous solution.

Rumex abyssinicus is a plant species with diverse nature and properties that have attracted attention in recent studies. This plant species belongs to the genus Rumex, which encompasses approximately 975 species, with only 200 of them having recognized scientific names [34]. Rumex plants are widely distributed across various regions, including Africa, Asia, Europe, North America, and Australia. Rumex abyssinicus is known for its high biomass production, resilience to harsh environmental conditions, and its abundance in various regions [[35], [36], [37], [38]]. The unique characteristics of Rumex abyssinicus have led to its exploration for various applications. One notable application is the production of activated carbon derived from Rumex abyssinicus biomass. Activated carbon obtained from this plant has shown promising adsorption properties due to its porous structure and high surface area [31]. The effectiveness of Rumex abyssinicus derived activated carbon has been demonstrated in the removal of various contaminants from water and wastewater, including methylene blue [35], Brilliant Blue reactive [36], Malachite green [38] and reactive Black MNN [37]. The adsorption capacity and efficiency of this activated carbon make it a potential candidate for water treatment and environmental remediation applications. However, the comprehensive cost analysis of Rumex abyssinicus based activated carbon has evidenced about 98 % of cost associated with activated carbon preparation is associated with chemical activation [36]. Hence, unmodified Rumex abyssinicus stem based biosorbent was selected to tackle the cost associated with thermal and chemical activation. To the best of authors’ knowledge, no research has been done on utilization of un-modified Rumex abyssinicus stem based biosorbent for pollutant detoxification. Moreover, the adsorption of methyl orange onto Rumex abyssinicus based biosorbent material is missing. To this end, this research is aimed to investigate the potential of Rumex abyssinicus based powdered unmodified biosorbent material for methyl orange decolorization from aqueous solution. Additionally, the isotherm, kinetics and thermodynamics and regeneration and reusability studies have been undertaken in order to further investigate the mechanism as well the feasibility of the adsorption process.

## 2 Materials methods

### 2.1 Biosorbent preparation

The powdered biosorbent material was prepared using Rumex abyssinicus stems as a precursor material. Precisely, the sun-dried stem of Rumex abyssinicus was collected from Addis Ababa, Ethiopia. The plant specimen was obtained by the researchers (Ashagrie Mengistu and Mikiyas Abewaa) and cross-checked against the herbarium of Addis Ababa University, where the voucher plant specimen (without an ID) was deposited. Then, the collected material was thoroughly washed with distilled water after being rinsed using distilled water in order to remove impurities adhered to its surface. Then, the dust and impurities freed sample was subjected to overnight oven drying (105 °C for 24 h), in order to make the precursor material completely crispy. Thereafter, the oven dried material was pulverized and sieved to particle sizes of 250 −150μm. Finally, the powdered biosorbent material prepared from Rumex abyssinicus stem was stored in an airtight plastic container. All procedures were carried out in conformity with pertinent institutional, national, and international laws and regulations.

### 2.2 Biosorbent characterization

#### 2.2.1 Scanning Electron microscope (SEM) analysis

Various surface morphologies, porosity formation, and aggregates expected on the adsorbent were studied using SEM analysis at Adama Science and Technology University. Sample preparation and scanning were performed according to the procedures of the machine manual and its operating procedures. Aluminum specimen stubs with double-sided SEM were used to attach the biosorbent powder and sputter-coated with a thin layer of gold. Imaging was done with a 10 μm resolution and a guaranteed capacity of 10 kV with a model JCM-6000PLUS BENCHTOP SEM (JOEL), Japan at 10 kV in the secondary electron imaging mode and coupled to an Imix-I digital image workstation [39].

#### 2.2.2 Fourier Transform Infrared (FTIR) spectroscopy analysis

Functional group identification is important to know the functional groups involved and predict the expected interactions between the adsorbent and adsorbate. FTIR spectroscopy technique was used to analyze functional groups of the prepared biosorbent product at Leather and Leather Products Research and Development Center, where the FTIR spectrum was recorded in the region of 4000 to 400 cm^−1^. The functional groups were determined using an FTIR spectrophotometer (SHIMADZU IR Affinity-1S). The adsorbent powder was mixed with dry KBr at a ratio of 1:100, and then the mixture was ground into a fine powder using mortar and pestle to make it suitable for infrared analysis. Finally, the fine powder was made into a pellet 1.5 mm thick under a load of 8 tons press. Then, the prepared pellet was placed in the sample holder. The spectrum was scanned in a transmission mode under a scanning rate and resolution of 32 times/min and 4 cm^−1^, respectively [[40], [41], [42]].

#### 2.2.3 X-ray diffraction (XRD) analysis

Information related to the crystal structure and chemical compositions of the biosorbent material was studied using XRD which is a non-destructive analytical technique. The machine used for the analysis was an XRD instrument (XRD-X-ray tube cu 40 kv, 44 mA, Rugaku, Ultima IV), at the Leather and Leather Products Research and Development Center. The X-ray beams’ scattered intensity hitting the adsorbent sample versus the angle of incident and angle of scattering were used to conduct the task. It has an initialization power of 15 kV and 5 mA. A scan speed of 6 rev/min over an angle ranging from 5 to 80° was used to carry out the analysis in which the power was set to 40 kV and 44 mA [43,44].

#### 2.2.4 Determination of point of zero charges

Mass titration and acid titration methods were used methods to determine the pH point of zero charges (pH_pzc_). Normally, at the pH point of zero charge, the net charge on the total surface of the sample becomes zero due to equal amounts of negative and positive charges. In this particular study, a mass addition method was used where 50 mL of 0.1 M NaCl was prepared in six 250 mL Erlenmeyer flasks and the pH of which was adjusted varying between 2 and 12 using 0.1 M HCl or 0.1 M NaOH. Then, 1g of the sample was added to each of the flasks and finally, after shaking at 125 rpm for 48h, the final pH of the solutions was determined using a digital pH meter, and the pHpzc was determined by drawing curves relating to the pH final and pH initial as the point where the two curves intersect [45].

### 2.3 Batch adsorption optimization

The Box Behnken approach to response surface methodology was used for the optimization of methyl orange adsorption in batch mode. A total of 30 experiments were conducted utilizing four operating parameters (pH, contact time, absorbent dosage and initial methyl orange concentration) where the removal efficiency is a response variable. These four operating parameters are designed at three levels; pH (3, 6 and 9), initial dye concentration (10, 20 and 30 mg/L), adsorbent dosage (0.1, 0.2 and 0.3 g/100 mL) and contact time (30, 60 and 90 min) as shown in Table 1. The designation of lower value (−), middle value (0) and higher value (1) were used to elucidate the symbolization of respective operating parameters levels and values.

**Table 1:** Box Behnken experimental design for methyl orange adsorption.

Parameter	Lower (−)	Middle (0)	Higher (+)
pH	3	6	9
**Contact Time (min)**	30	60	90
**Biosorbent dose (g/100 mL)**	0.1	0.2	0.3
**Initial dye concentration (mg/L)**	10	20	30

### 2.4 Methyl orange batch adsorption

Methyl orange batch adsorption was conducted in 100 mL of distilled water utilizing the design experiments. Initially, 0.01, 0.02 and 0.03 g of adsorbate were dissolved in 1 L distilled water separately, which gave a dye concentration of 10, 20 and 30 mg/L solution respectively. Then, 100 mL of the solution was transferred to Erlenmeyer flasks from each sample solution based on the design experiments. Then pH of the solution was adjusted based on the basicity or acidity of the solution using 0.1 M NaOH and 0.1 M HCl. Afterwards, the desired amount of adsorbent material was added to the solution and the solution was shaken at 125 rpm for a specified duration of contact time, taking the experimental design into account. Finally, as a contact time expired, the supernatants were filtered using Whatman filter paper and the filtrate was used for the determination of the amount of dye remaining in solution after adsorption [46]. The analytical determination of residual methyl orange left unadsorbed was carried out using UV VIS at a maximum wavelength of 507 nm. For this purpose, a calibration curve was developed using varied concentrations of adsorbate (5, 10, 15, 20, 30 and 35 mg/L). Thereafter, Beer lambert equation was used to calculate the unknown dye concentration remaining in the solution as shown in the supplementary material. Finally, removal efficiency (RE) and adsorption capacity (Qt) were determined using Equations (1), (2)) respectively.

(1) $$\text{RE}\;\left(\%\right)=\;\frac{Co-Ct}{Co}\times 100$$

(2) $$\text{Qt}\;\left(\text{mg}/g\right)=\left(\frac{Co-Ct}{m}\right)\times V$$

where, Co and Ct initial and final dye concentration respectively, V denotes solution volume and m is mass of adsorbent utilized [[47], [48], [49]].

### 2.5 Adsorption isotherm

The effectiveness of the adsorption process can be modelled using several adsorption isotherms varying the initial dye concentration (10, 15, 20, 25 and 30 mg/L). However, other operating parameters were held at constant optimum values of pH, contact time and adsorbent dosage of 6, 60 min and 200 mg/100 mL respectively. Fundamentally, the dye concentration dependency of the adsorption system is aimed at investigating the nature, and mechanism of adsorption pertaining. There are two and three parameters adsorption isotherm models used in designing various adsorption systems. In this study, two parameter adsorption isotherms (Langmuir and Freundlich) and three parameter isotherm models (Toth and Koble Corrigan) were used to evaluate the dye adsorption nature at equilibrium. Consequently, Langmuir (equation (3)), Freundlich (equation (4)), Toth (equation (5)) and Koble-Corrigan (equation (6)) were used for analysis of isotherm studies of methyl orange adsorption from an aqueous solution through Rumex Abyssinicus derived biosorbent as shown in Table 2.

**Table 2:** Adsorption isotherm models used for evaluation of methyl orange adsorption.

Model	Equation Equation number	Reference
**Langmuir**	(3) $\frac{QmaxKLCe}{1+KLCe}$	[50]
**Freundlich**	(4) $\text{Qe}={\text{KFCe}}^{1/\text{n}}$	[51,52]
**Toth**	(5) $\text{Qe}=\frac{Q_{\max}KCe}{{\left[1+{\left(KCe\right)}^{nt}\right]}^{\frac{1}{nt}}}$	[53,54]
**Koble Corrigan**	(6) $\text{Qe}=\frac{A{C_{e}}^{nk}}{\left(1+B{C_{e}}^{nk}\right)}$	[55,56]

Additionally, the adsorption dimensionless factor constant (RL) that is used to estimate Langmuir isothermal feasibility is indicated by equation (5).

(7) $$\text{RL}=\frac{1}{1+K_{L}\text{Ce}}$$

### 2.6 Adsorption kinetics

Models for pseudo-first order, pseudo-second order, and intraparticle diffusion were used to assess the adsorption kinetics. In general, adsorption kinetics investigations are paramount important since they reveal details about the rate of dye absorption and the adsorption process' mechanism. Additionally, kinetics studies are assessed to establish if chemosorption or physcosorption occurs during the interaction of the adsorbent and adsorbate. Basic nonlinear kinetics equations are shown in Table 3, and these equations are then utilized to calculate the constants associated with each kinetics model. The initial dye concentration, adsorbent dose, and contact time of the process were all kept at their optimal levels of 6, 0.2 g/100 mL, and 20 mg/L, respectively, for the kinetics investigations.

**Table 3:** Adsorption kinetics models applied for methyl orange adsorption.

Kinetic model	Kinetics equation Equation number	References
**Pseudo first order**	(8) $\text{Qt}=\text{Qe}\left(1-e^{-K1t}\right)$	[57,58]
**Pseudo second order**	(9) $\text{Qt}=\frac{{Qe}^{2}K_{2}t}{1+QeK_{2}t}$	[58,59]
**Intraparticle diffusion**	(10) $\text{Qe}={kd\times t}^{0.5}+C$	[60,61]

### 2.7 Adsorption thermodynamics

The ideas related to energy and feasibility of the adsorption process are evaluated using adsorption thermodynamics by varying the temperature of the system while holding all the operating parameters at their respective optimum values (pH of 6, contact time of 60 min, adsorbent dosage of 200 mg/100 mL and dye concentration of 20 mg/L). During the thermodynamics study of the adsorption of methyl orange onto Rumex abyssinicus derived biosorbent material, the temperature of the system varied from 25 to 65 °C (25, 35, 45.55 and 65°C). Change in Gibbs free energy ($\Delta G^{O}$), Enthalpy change ($\Delta H^{O}$) and Entropy change ($\Delta S^{o}$) were determined using Van't Hoff equation given by equations 11, 12, 13, 14). Where, R is the universal gas constant (8.314 J/mol. K), KC represents the thermodynamic constant, and T is the absolute temperature (K). Ce denotes the amount of MB dye concentration at equilibrium; qe represents the amount of dye adsorbed on the adsorbent at equilibrium (mg/g) [62,63].

11 Δ***G*** = −***RTlnKC***

12 $$\text{KC}=\frac{qe}{Ce}$$

13 $$\ln KC=\frac{\Delta S}{R}-\frac{\Delta H}{RT}$$

14 Δ***G*** = Δ***H*** − ***T***Δ***S***

## 3 Results and discussions

### 3.1 Adsorbent characteristics

#### 3.1.1 Results of pHpzc analysis

**Fig. 1:**
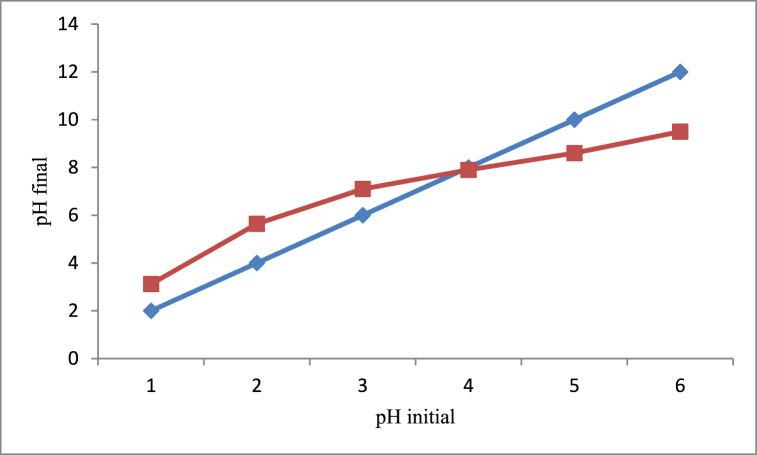
pHpzc of powdered Rumex abyssinicus biosorbent material.

The pHpzc of rumex Abyssinicus derived unmodified adsorbent material was determined to be 7.9 as shown in Fig. 1. Basically, the surface of the biosorbent material is dominated by negative charges above the pHpzc and positively charged below the pHpzc. Furthermore, at pHpzc the positively and negatively charged surfaces of the adsorbent become equal showing a neutral charge. In this regard, cationic dyes are sufficiently adsorbed at the pH of the surface above the pHpzc and an ionic dye like methyl orange adsorption is favoured at positively charged surfaces. The pHpzc of currently produced biosorbent material is nearly higher than the activated carbon produced from the same material reported by the same researchers such as 7.2 [37], pHpzc of 5.1 [38], pHpzc of 6.9 [36] and pHpzc of 5.03 [35]. This is due to the fact that the phosphoric acid applied for activating the adsorbent material in case of activated carbon could make it mildly acidic thereby decreasing its pHpzc. On the other hand, there is a huge opportunity for maximum adsorption of ionic dyes since the surface of the biosorbent positively charged is higher compared to negatively charged surfaces.

#### 3.1.2 SEM-surface morphology analysis result

**Fig. 2:**
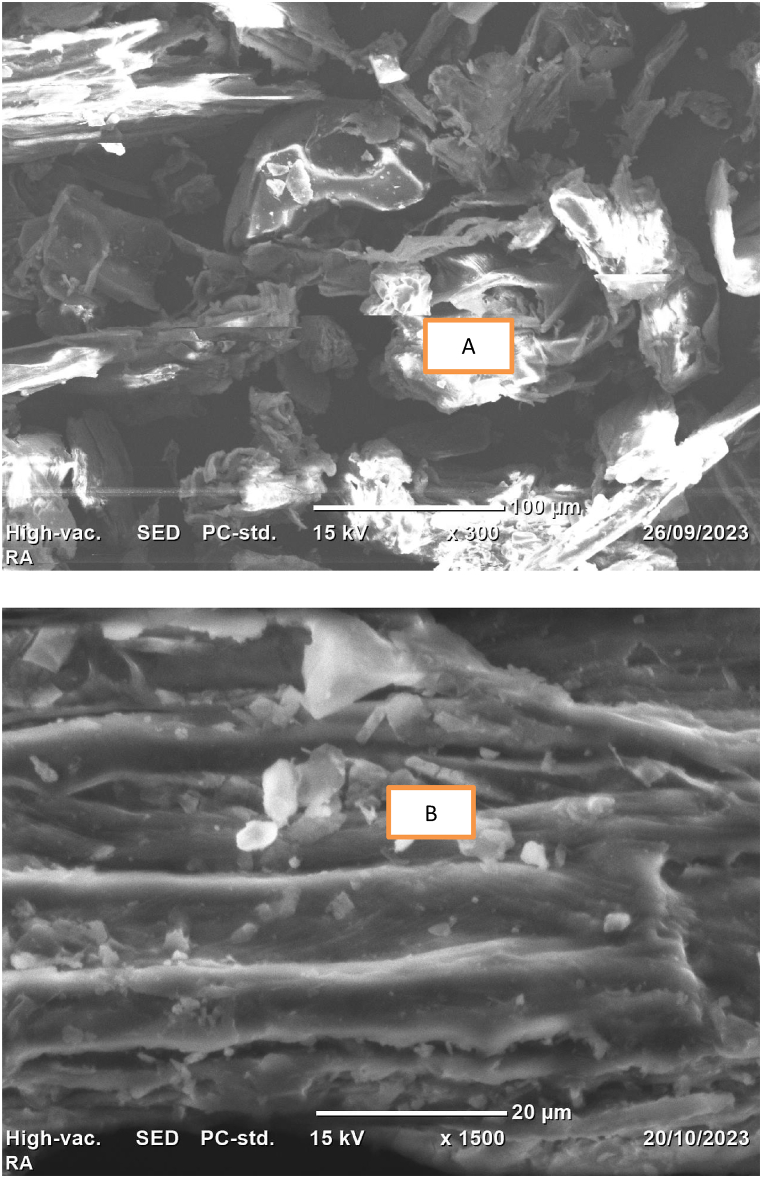
SEM micrographs of Rumex abyssinicus derived biosorbent material.

The SEM was used to assess the surface morphology of the biosorbent material. As a result, Fig. 2A shows that the biosorbent material's surface is highly irregular, porous, and heterogeneous before adsorption. However, more regular morphology with less cavity and holes were observed after adsorption as shown in Fig. 2B. This indicated the successful attachment of the methyl orange onto the porous structure of the adsorbent decreasing the porosity as well as cavity. Generally, the SEM analysis revealed that the biosorbent under consideration is composed of a material that is suitable for adsorbing pollutants onto their pores. This result is consistent with earlier research that was mentioned by Refs. [[35], [36], [37], [38],^64^].

#### 3.1.3 XRD analysis

**Fig. 3:**
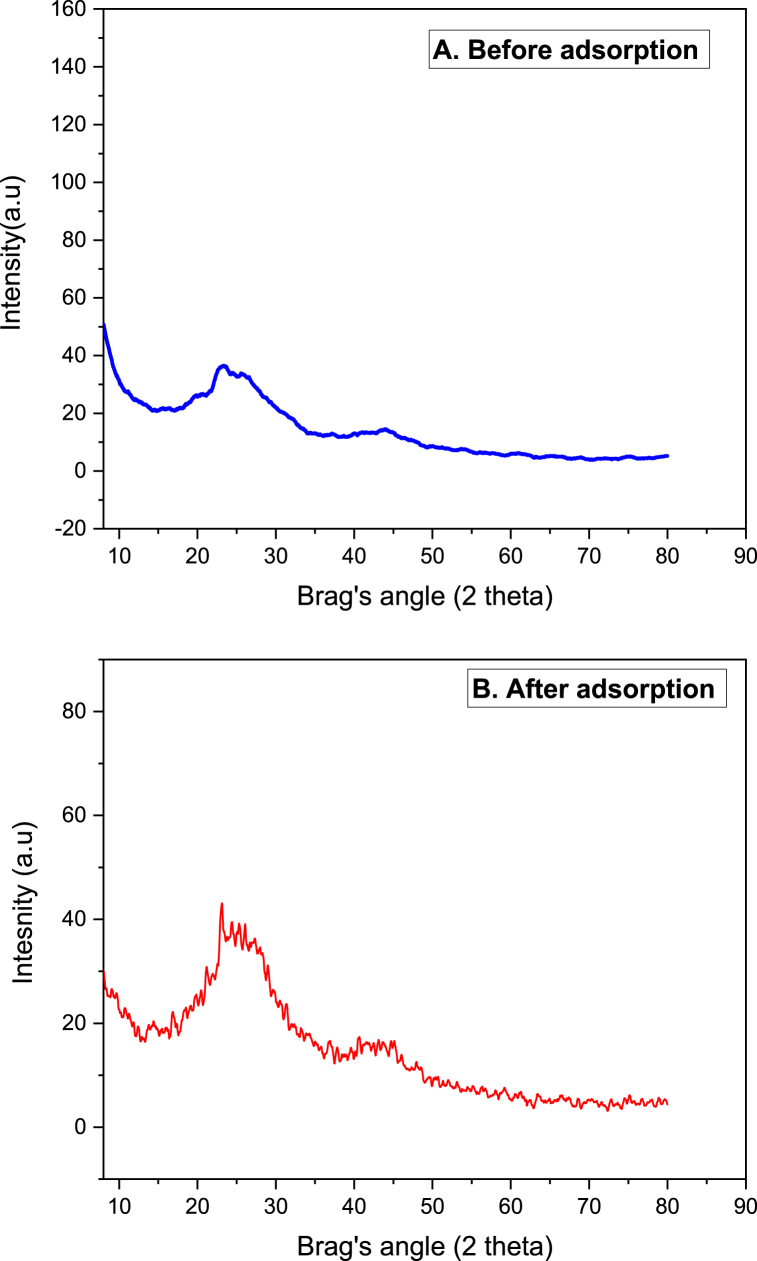
XRD for Rumex abyssinicus derived biosorbent: before adsorption (A) and after adsorption (B).

The crystalline nature of the adsorbent material is investigated using XRD and the result of the study is shown in Fig. 3a and b. As it can be observed from the Figure, the overall structure of the biosorbent material is found to be amorphous which is a typical structure of biomass based biosorbent materials, however, the peak on (2θ of 24°) has a particular location where the crystalline character of the biosorbent material was observed. This is attributed to the cellulose constitute of the material [65]. Normally, amorphous surfaces exhibit higher adsorption capability compared to crystalline structure having materials. This is due to the fact that crystalline surfaces are highly ordered which needs energy to penetrate the crystal structure.

#### 3.1.4 FTIR analysis

**Fig. 4:**
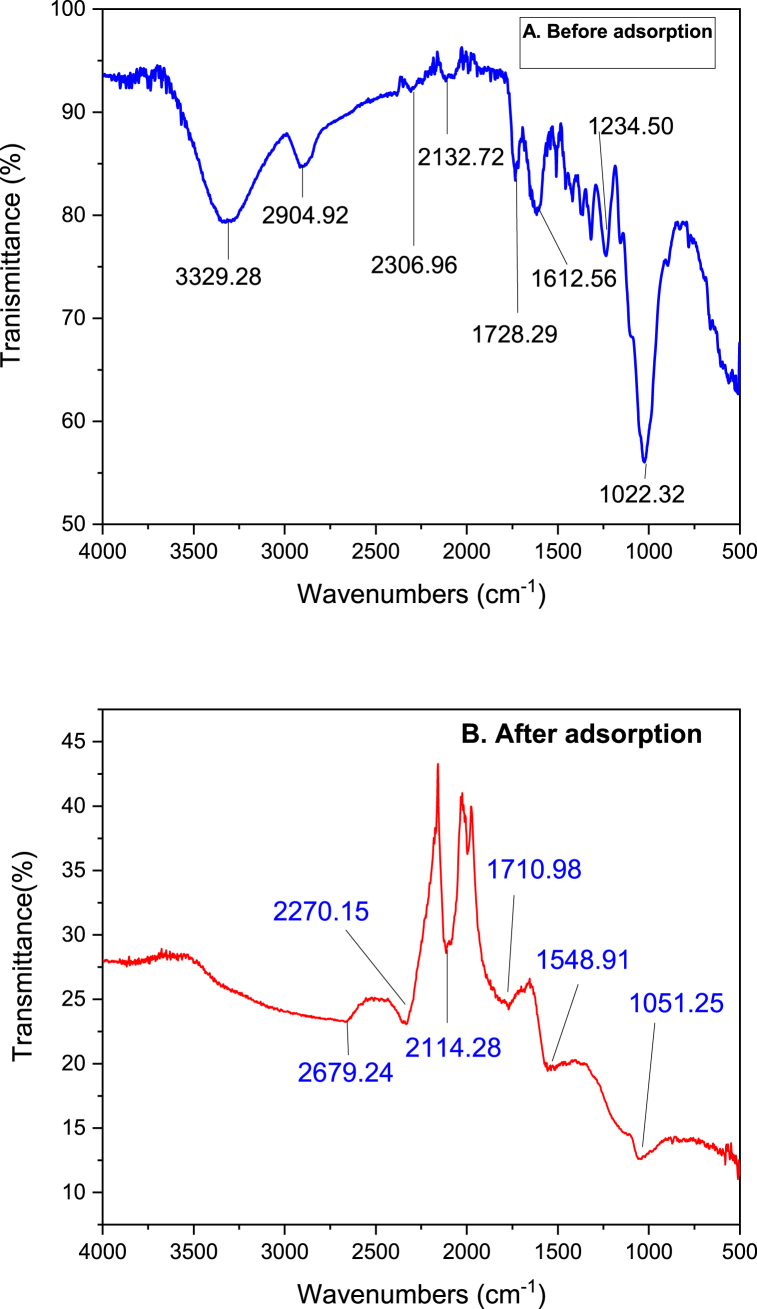
FTIR analysis result of Rumex abyssinicus biosorbent before adsorption (A) and after adsorption (B).

The FTIR spectra of the biosorbent of Rumex abyssinicus stem in the range of 400–4000cm−1 were taken to obtain information about functional group interactions as shown in Fig. 4. The infrared spectrum exhibited broad bands centred at 3329.28 cm^−1^ assigned to O–H and N–H stretching, the stretching vibration of C=O at 1612 cm^−1^ and C–O stretching of alcoholic groups at 1022.32 cm^−1^. The peak at 2904.92 cm^−1^ is attributed to the O–H and C–H stretching vibrations of carboxylic acid, alcohol, or alkene groups. On the other hand, The clear sharp peaks located at 1728.29 cm^−1^ indicates the existence of C=O or C=C stretching vibration of acid derivatives, which are characteristics of carbonyl group stretching from aldehyde and ketones [66]. On the other hand, the peak observed at 2132.72 cm^−1^ would indicate the stretching vibration of alkyne group. The strong starching vibration observed at 1612.56 cm^−1^ is attributed to C=C bond. The characteristics of C=N bond is indicated by the peak observed at 2306.96 cm^−1^. On the other hand, diminishing and shifting of peaks were observed after the pollutant loaded on to the rumex Abyssinicus derived biosorbent material. The major peaks observed after adsorption were determined to be 2679.24 cm^−1^, 2270.15 cm^−1^(C=N), 2114.28 cm^−1^(Stretching of alkene group), 1710.68 cm^−1^(C=O), 1548.91 cm^−1^(C=O) and 1051.25 cm^−1^(C–O). Generally, the formation, diminishing and shifting of peaks observed after adsorption indicates the interaction of methyl orange with Rumex abyssinicus derived biosorbent material [[67], [68], [69]].

### 3.2 Batch adsorption performances

The removal efficiencies of methyl orange onto powdered Rumex abyssinicus based biosorbent ranged from 24.6 to 98.5 % as shown in Table 4.

**Table 4:** Batch adsorption performances of methyl orange adsorption onto Rumex abyssinicus biosorbent.

Run No.	pH	**Dye con. (mg/L)**	Adsorbent dosage (g/100 ml)	Contact time (min)	Removal efficiency (%)
**1**	3	20	0.3	60	89.65
**2**	9	10	0.3	60	59.24
**3**	3	10	0.1	60	81.87
**4**	3	20	0.2	60	73.43
**5**	6	30	0.3	60	68.81
**6**	6	20	0.1	60	82.95
**7**	3	10	0.1	60	76.34
**8**	9	10	0.2	60	72.86
**9**	6	20	0.3	60	63.47
**10**	9	20	0.1	60	55.54
**11**	3	20	0.2	30	91.40
**12**	3	20	0.3	30	94.26
**13**	6	20	0.2	30	93.79
**14**	3	30	0.2	30	84.38
**15**	9	30	0.1	30	24.61
**16**	6	10	0.1	30	97.06
**17**	6	20	0.2	30	95.06
**18**	6	30	0.3	30	90.24
**19**	3	20	0.1	30	83.62
**20**	6	30	0.2	30	87.45
**21**	9	10	0.3	90	65.85
**22**	3	20	0.1	90	78.68
**23**	9	30	0.3	90	85.48
**24**	9	10	0.2	90	90.21
**25**	9	30	0.1	90	63.24
**26**	9	10	0.1	90	81.14
**27**	9	30	0.3	90	88.64
**28**	6	30	0.3	90	86.47
**29**	3	10	0.1	90	93.19
**30**	6	30	0.2	90	79.08

By exhibiting significant variation elimination efficiency over a range of operational parameters, this study illustrated the important impact of the experimental variables. The maximum removal rate of 98.5 % was achieved under experimental circumstances of pH 6, contact time of 60 min, starting dye concentration of 20 mg/L, and adsorbent dosage of 0.2 g/100 mL. The lowest removal effectiveness (24.61 %) found in this experiment was reported at treatment circumstances of adsorbent dosage of 0.1 g/100 mL, contact length of 30 min, starting dye concentration of 30 mg/L, and pH of 9. As shown in Table 5, the best removal efficiency observed in the current experiment is promising and reasonably priced when compared to other biomass-based adsorbents employed for the removal of methyl orange. Precisely, Husien, S. et al. investigated the application of fava bean peel based activated carbon for the removal of methyl orange and reported 96.8 and 94 % adsorption efficiency for thermally and chemically activated biomass respectively [70]. In similar manner, Jackfruit leaves derived un activated biosorbent was able to remove 78.1 % of methyl orange [71]. On the other hand, Abdisa, A. et al. reported the maximum removal of 94. 47 % for anchote peel waste based biosorbent material applied for the removal of methyl orange from aqueous solution [72]. These studies suffer from low removal efficiency. Hence, the Rumex abyssinicus derived activated carbon can be taken as alternative raw material for dye detoxification with effective adsorption performance.

**Table 5:** Comparative study for different biomass based adsorbents applied for methyl orange adsorption.

Adsorbent	Activation method	Removal efficiency/adsorption capacity	References
**Powdered Rumex abyssinicus**	No activation	98.5 %	This study
**Raw fava bean peels**	Thermal activation(at 600 °C)	96.8 %	[70]
**Raw fava bean peels**	Chemical activation (H3PO4)	94 %	[70]
**Jackfruit leaves**	No activation	78.10 %	[71]
**populous leaves**	No activation	17.2 mg/g	[73]
**Populous leaves**	Chemical activation(acetic acid)	40.5 mg/g	[73]
**Coconut Shell Activated Carbon**	Thermal activation(500 °C) and chemical activation (ZnCl_2_)	59.17 mg/g	[74]
**Tamarind seed activated carbon**	Thermal activation (700 °C) and chemical activation (H3PO4)	94.45 mg/g	[75]
**Brachychiton populneus**	Chemical activation (NaOH and FeCl_3_)	955	[76]
**Corncob**	Chemical activation (H3PO4)	99.75 %	[77]
**Ocimum basilicum Linn Leaves**	Chemical activation (H2SO4) and Thermal activation (500 °C)	1.54 mg/g	[78]
**tamarind shell activated carbon**	Chemical activation(NH4Cl) and thermal activation (500 °C)	24.3 mg/g	[79]
**candlenut shell (Aleurites moluccana)**	Chemical activation(HNO3 and H3PO4) and thermal activation(700 °C)	4.80 mg/g	[80]
**Orange and lemon peel activated carbon**	Chemical activation(H3PO4) and thermal activation (600 °C)	33 mg/g	[81]
**Date seeds**	Chemical activation(H3PO4) and thermal activation(500 °C)	99.82 %	[82]
**Anchote peel**	No activation	94.47 %	[72]

### 3.3 Effects of operating parameters

#### 3.3.1 Effect of contact time

**Fig. 5:**
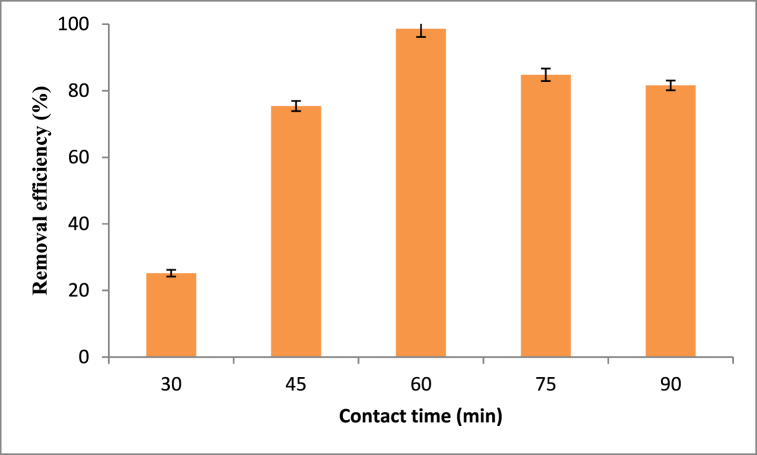
Effect of contact time on methyl orange adsorption

To investigate the effect of contact time for the adsorption of methyl orange onto Rumex abyssinicus based biosorbent, an experiment was done for a contact time of 30–90 min. During the effect of contact time study, the pH of the solution, adsorbent dosage and initial dye concentration were kept constant at their respective values of 3, 20 mg/L and 0.3 g/100 mL. Moreover, the effect of contact time was analysed at a fixed agitation speed of 125 rpm to observe the contact time variation effect on the adsorption performance exclusively. As shown in Fig. 5, the removal efficiency of methyl orange increases as contact time increases until it reaches the equilibrium point. At a shorter contact time of 30 min, the removal efficiency was 25.2 %. Meanwhile, the maximum removal efficiency of 98.6 % was attained at a contact time of 60 min. Further increase in contact time beyond 60 min did not show an increase in removal efficiency rather slightly decreased the removal efficiency signifying the attainment of equilibrium point. This is most likely because there isn't an active site for the methyl orange dye ions to be adsorbed onto the biosorbent material made from Rumex abyssinicus, and the driving force is decreasing [83].

#### 3.3.2 Effect of pH

**Fig. 6:**
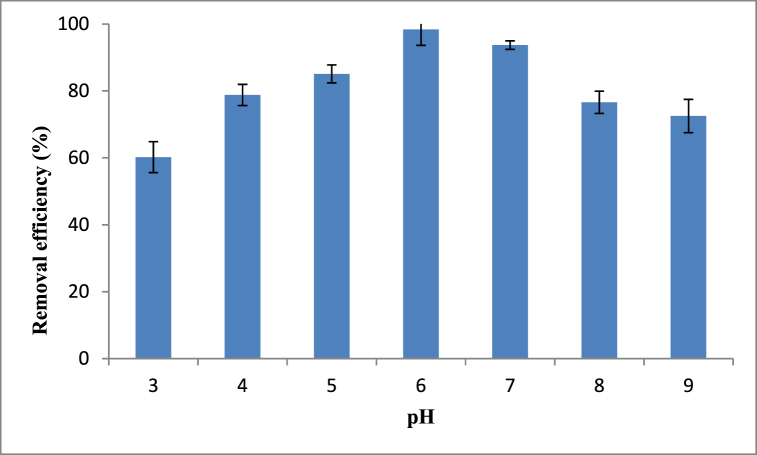
Effect of pH on Methyl orange removal efficiency

The effect of pH on sorption of anionic dye, methyl orange was studied at seven different levels of pH varying from 3 to 9 with an increment in1. The effect of pH on the extent of sorption of methyl orange is shown in Fig. 6. It can be observed that the maximum amount of methyl orange was adsorbed at pH 6 with a percentage removal efficiency of 98.4 %. Furthermore, the removal efficiency declined dramatically as the pH of the solution increased from 6 to 9. This can be justified by the fact that above pH of 6, hydroxide ions increment causes a decrease in the adsorption of dye molecules at the adsorbent adsorbate interface. Notably, as the amount of hydroxide ions increases, it competes with anionic methyl orange for available active sites thereby decreasing removal efficiency [[84], [85], [86]]. Compared to the negative surface charges of the adsorbent, the higher removal efficiency of methyl orange was recorded at positive surface charges (pH < pHpzc). For instance, the maximum removal efficiency recorded at pH of 6 supports the pHpzc concept in which it is usually favorable to adsorb anionic dyes like methyl orange over the range of positively charged surface charges (0–7.9). This is due to the fact that at pH > pHpzc the anionic methyl orange dye competes with OH- groups hindering the adsorption efficiency hence pH < pHpzc is creates conducive environment for the adsorption process [[87], [88], [89], [90]]

#### 3.3.3 Effect of adsorbent dose

**Fig. 7:**
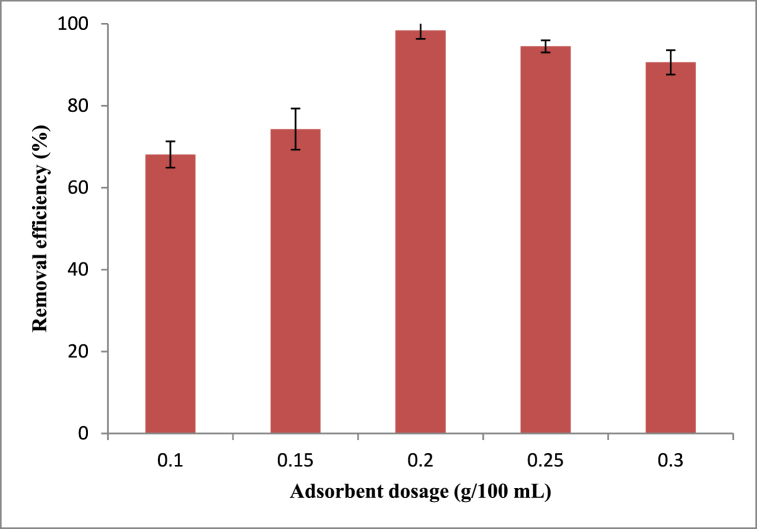
Adsorbent dosage effect on methyl orange removal efficiency

The effect of the adsorbent dose on the removal efficiency of methyl orange onto Rumex abyssinicus stem-based biosorbent material was examined over the biosorbent dose ranging from 0.1 to 0.3 g/100 mL. During the effect of adsorbent dosage investigation on the removal efficiency of methyl orange, the experimental conditions were fixed at pH of 6, contact time of 60 min and initial dye concentration of 20 mg/L. As shown in Fig. 7, the removal efficiency of methyl orange increased from 68.1 to 98.4 % as the adsorbent dose increased from 0.1 to 0.3 g/100 mL. The increase in methyl orange removal efficiency with an increase in adsorbent dose is obviously due to the increase of adsorption sites. However, a further increase in the adsorbent dosage results in a slight decline in removal efficiency showing the attainment of equilibrium point. On the other hand, the adsorption capacity of Rumex abyssinicus stem-derived biosorbent decreased as the dosage increased unlike that of removal efficiency. This is because of the unsaturated adsorbent active sites as the mass of the adsorbent kept increasing for constant initial dye concentration.

#### 3.3.4 Effect of initial dye concentration

**Fig. 8:**
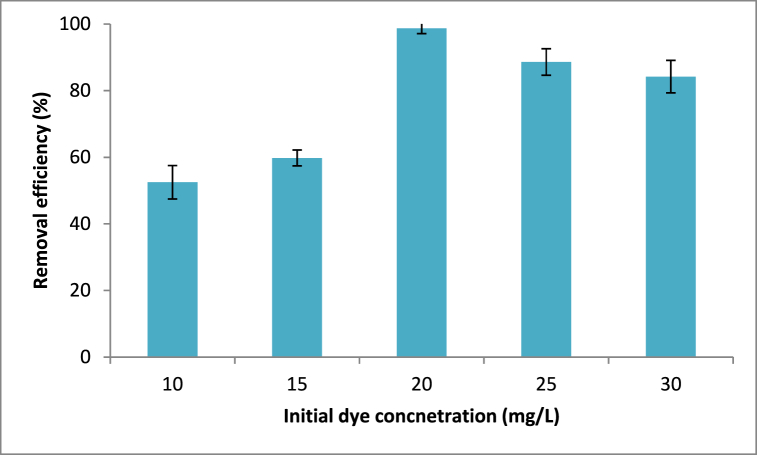
Initial methyl orange concentration effect on removal efficiency

At various concentrations of 10–30 mg/L with increments of 5 mg/L, the impact of the initial dye concentration on the degree of dye uptake was investigated. The other operating parameters, aside from initial dye concentrations, are maintained at their respective values of pH 6, contact period of 60 min, and adsorbent dosage of 0.2 g/100 mL. Fig. 8 demonstrates that as the initial dye concentration increased, so did the removal efficiency of the dye molecules from Rumex abyssinicus adsorbent material. As the initial dye concentration increased from 10 to 20 mg/L, the elimination efficiency rose from 52.5 % to 98.7 %. The interaction between the dye molecules and the surface of the adsorbent is often enhanced by an increase in the initial concentration of dye molecules. This can be explained by the fact that a higher concentration of dye molecules provides a driving force to overcome mass transfer resistance between the adsorbent and adsorption medium. However, increasing the initial methyl orange concentration above 20 mg/L has resulted in the decline of the removal efficiency because more dye molecules are left unabsorbed in the solution due to the saturation of binding sites. Fundamentally, for low dye molecule concentrations, the ratio of an initial number of moles of these molecules to the available surface area of the adsorbent is large and subsequently, the fractional adsorption becomes independent of initial dye concentration. However, at higher concentrations, the available sites for the adsorption become fewer, and hence the percentage removal efficiency of dye molecules depends up on the initial concentration [91,92,92].

### 3.4 Adsorption isotherm analysis result

**Fig. 9:**
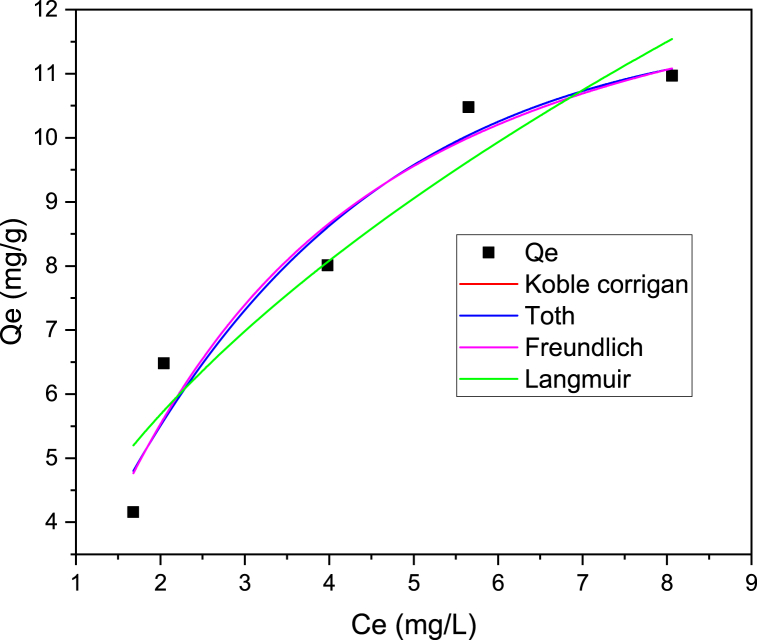
Adsorption isotherm analysis result for removal of methyl orange onto Rumex abyssinicus biosorbent

The adsorption isotherm for methyl orange removal was investigated where the experimental results obtained were introduced to the Langmuir, Freundlich, Toth and Koble Corrigan isotherm models. These isotherm models’ parameters were determined from the plot of Ce vs Qe as shown in Table 6. Furthermore, the respective nonlinear plots for Langmuir, Freundlich, Toth and Koble Corrigan isotherm models are given in Fig. 9. The coefficient of determination (R^2^) of Freundlich model for the adsorption of methyl orange was 0.94628, which is slightly higher than correlation coefficients of Langmuir (0.94428), Koble Corrigan (0.94528) and Toth (0.94486). This implies the adsorption of methyl orange onto powdered Rumex abyssiniccus stem well fitted to the Freundlich model, inferring the Rumex abyssinicus adsorbent material surfaces are heterogeneous sorption patches. Freundlich isotherm model constant parameters, KF (3.992) and n (1.97) were determined from non-linear equation plot Freundlich isotherm model. The parameter KF of this isotherm determines the degree of adsorption; low KF value indicates minimal adsorption whereas higher KF value implies greater sorption ability. Accordingly KF value obtained for the Methyl orange adsorption was 3.99, which implies favorable condition according to the model. Adsorption intensity, n from Freundlich isotherm was determined in this study which used to show the concentration of solute adsorption. The value of 1/n closer to 1 shows just a little concentration change can relatively affect the adsorption [93]. The value of n greater than 1 implies the adsorbent can effectively adsorb the solute. From the Freundlich isotherm model equation the values of n obtained was 1.97 and 1/n was 0.50825. Since the value of n is greater than 1 and 1/n lies between 0 and 1, the Rumex abyssinicus stem based biosorbent material can adsorb methyl orange effectively. Generally, owing to its highest coefficient of determination and insignificant reduced chi square the Freundlich isotherm term was found to effectively fit the plot best inferring heterogeneous and multilayer adsorption.

**Table 6:** Adsorption isotherm parameters values.

Adsorption isotherm model	Parameters
**Freundlich**	R^2^ = 0.946 KF = 3.99 1/n = 0.508 n = 1.97 Reduced Chi-square = 0.8942
**Langmuir**	R^2^ = 0.944 KL = 3.69 RL = 0.61 Qmax = 13.36 Reduced Chi-square = 0.87625
**Koble Corrigan**	R^2^ = 0.945 A = 3.6094 B = 0.27033 nk = 1.38662 Reduced Chi-square = 0.87625
**Toth**	R2 = 0.944 Qmax = 97.44 nt = 1.369 K = 0.16062 Reduced Chi Square = 0.88296

### 3.5 Adsorption kinetics study results

Table 7 shows the adsorption kinetics parameters of removal of methyl orange onto Rumex abyssinicus adsorbent. In this study, pseudo first order, pseudo second order and Intraparticle diffusion models were investigated. The nonlinear curve fit of t vs Qt was used to determine the basic parameters used to illustrate the nature of methyl orange adsorption onto Rumex abyssinicus adsorbent. Accordingly, the pseudo second order with maximum R^2^ 0.97 was found to fit data best. Moreover, the adsorption kinetics parameters associated with pseudo second order was found to be K_2_ (g/mg min) = 0.82, Qe cal = 14.62 mg/g and Reduced chi square was determined to be 0.1856 as shown in Table 7. On the other hand, pseudo first order's parameters such as R^2^, K1, qmax and Reduced chi square were determined to be 0.88, 2.21min^−1^, 16.41 mg/g and 0.61 respectively. The effect of diffusion during the adsorption of methyl orange onto Rumex abyssinicus based biosorbent was evaluated by employing the intraparticle diffusion model. Accordingly, the coefficient of determination, Kd, C and reduced chi square were found to have a value of 0.95, 3.15, 0.32 and 0.74 respectively. These results indicated that the intraparticle diffusion model was not a sole kinetics that controls the adsorption model nevertheless it has participated in the adsorption processes [94,95]. Generally, the adsorption kinetics study analysis revealed that the nature of the nature of the adsorption to be chemosorption onto heterogeneous surfaces of adsorbate-adsorbent interface.

**Table 7:** Kinetic parameters for adoption of methyl orange onto Rumex abysiniccus adsorbent.

Adsorption kinetic models	Respective kinetic parameters
**Pseudo first order**	R^2^ = 0.88 K1 = 2.21 Qcal = 16.41 Reduced chi square = 0.61
**Pseudo second order**	R^2^ = 0.97 K2 = 0.82 Qe cal = 14.62 Reduced chi square = 0.1856
**Intraparticle diffusion model**	R^2^ = 0.95 Kd = 0.32 C = 3.15 Reduced chi square = 0.74

### 3.6 Thermodynamics study

**Fig. 10:**
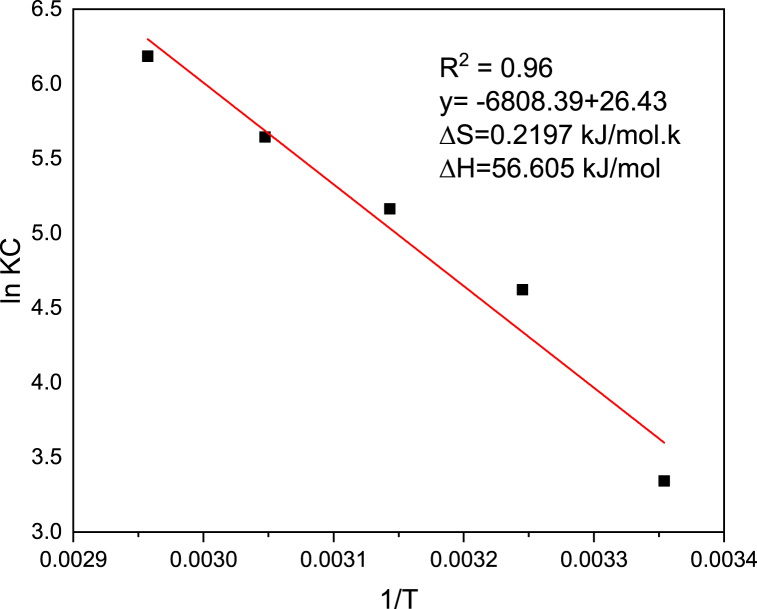
Adsorption thermodynamics for the removal of methyl orange onto *Rumex abyssinicus* biosorbent

Thermodynamics study of the methyl orange adsorption onto Rumex abyssinicus based biosorbent material was intended to determine the basic parameters that are used to give a clue to know whether the adsorption process is either feasible ($\Delta G^{O}$ <0) or not ($\Delta G^{O}$ >0), endothermic ($\Delta H^{O}$ >0) or exothermic ($\Delta H^{O}$ <0), spontaneous ($\Delta G^{O}$ <0) or non-spontaneous ($\Delta G^{O}$ >0) [96]. The thermodynamics graph that was used to evaluate the nature of the adsorption process was developed by plotting $\frac{1}{T}$ (X coordinate) against ln KC (Y coordinate) as shown in Fig. 10. Then, $\Delta H^{O}$ and $\Delta S^{O}$ were calculated from the intercept and slope of the plot using equation (13). Accordingly, $\Delta H^{O}$ and $\Delta S^{O}$ were determined to be 56.605 kJ/mol and 0.2197 kJ/mol.k respectively which in turn gave a negative $\Delta G^{O}$ value for all varied temperatures throughout the thermodynamics study as shown in Table 8. Moreover, it was found that Gibbs free energy change was determined to decline with increasing the temperature which is in agreement with spontaneous change. Normally, endothermic processes have a positive sign for enthalpy change, and raising temperature will result in a reduction in the Gibbs free energy change and make the process easier to carry out. On the other hand, the positive $\Delta S^{O}$ value determined in this work indicates the heterogeneity of the adsorption process supporting the adsorption isotherm finding. The range of temperatures at which the adsorption process is practical is predicted by the optimal temperature (T^0^), where the standard Gibbs free energy change is zero [97,98]. In this work, the operating temperature must be greater than 257.65 k for the process to be spontaneous. Generally, the thermodynamics analysis revealed that the adsorption of methyl orange onto Rumex abyssinicus stem based biosorbent material is endothermic, spontaneous and feasible.

**Table 8:** Thermodynamic parameters values for adsorption of methyl orange onto Rumex abyssinicus biosorbent.

**Temperature (k)**	$\Delta G^{O}$**(kJ/mol)**
**298.15**	−8.2845
**308.15**	−11.8396
**318.15**	−13.6577
**328.15**	−15.3976
**338.15**	−17.3895

### 3.7 Adsorbent regeneration and reusability

**Fig. 11:**
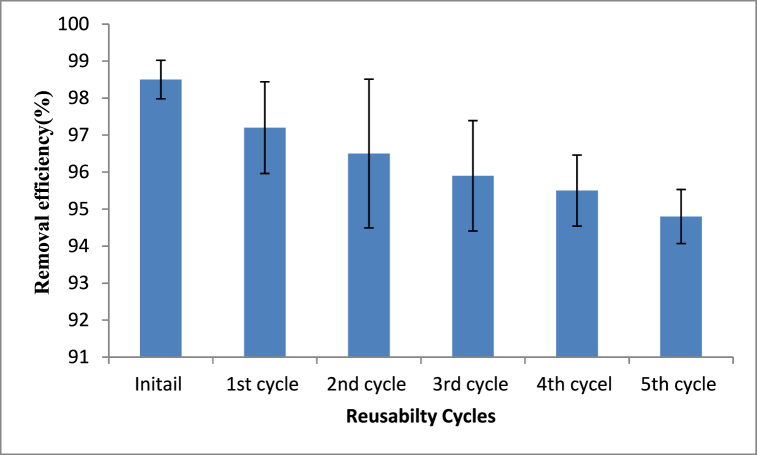
Reused Rumex abyssinicus biosorbent potential for removing methyl orange from aqueous solutions

Regeneration and reusing spent adsorbents are more economical than producing another adsorbent for pollutant detoxification. Moreover, regeneration and reusing of spent adsorbents have paramount environmental benefits as disposing of used adsorbents causes various environmental problems. Pollutant-loaded adsorbents are toxic to environmental segments like soil, water and air. Hence, the methyl orange loaded adsorbent material was regenerated using chemical methods following the same procedures indicated by Refs. [36,38,46]. The results of the reusability study have shown that the rumex abyssinicus stem-based biosorbent material has the potential to be reused for five cycles. As can be seen from Fig. 11, the removal efficiency of reused biosorbent material ranged from 97.2 to 94.8 % showing the effectiveness of rumex abyssinicus-based biosorbent for methyl orange decolorization.

### 3.8 Adsorption mechanism

Various characterization techniques such as FTIR, XRD and SEM as well as the isotherm and kinetics studies give a clue about the adsorption mechanism of methyl orange adsorption onto Rumex abyssinicus biosorbent. Normally, the determination of the adsorption mechanism is not straightforward due to the complex nature of the interaction between the adsorbate and adsorbent. The interaction of the pollutant and the adsorbent materials can be a weak physical force like dipole-dipole, electrostatic, covalent, non-covalent, or hydrogen bonding. More importantly, the nature of the adsorption is the synergic effects of both physical and chemical interactions. For this particular study, the isotherm study analysis revealed that the nature of the adsorption is multilayer signifying the existence of weak interaction like dipole-dipole forces whereas the kinetics study has shown the pseudo second order to fit the data best inferring the involvement of bond formation through chemisorption. Moreover, the covalent and non-covalent bonding (hydrogen bonding, ionic interaction andπ-π stacking) can be formed between the active functional groups present in the Rumex abyssinicus stem-derived biosorbent material (-OH, –C=C) and the target pollutant. On the other hand, methyl orange dye molecules can interact with –NH2 groups forming covalent and semi-covalent bonds. Additionally, the formation peaks like 2679.24 cm^−1^, the diminishing of hydroxide groups (3329 cm^−1^) as well as the shifting of various functional group signifying peaks like (2132–2114 cm^−1^, 1728 to 1710 cm^−1^, 2306 to 2270 cm^−1^, 1022 to 1051 cm^−1^) would indicate the interaction between the adsorbate and adsorbent where various functional groups played crucial role [82,[99], [100], [101]].

## 4 Conclusion

This study proposed an untreated powdered stem of Rumex abyssinicus as a low-cost and effective adsorbent for the removal of methyl orange from an aqueous solution. Accordingly, the characterization of the Rumex abyssinicus-derived biosorbent material has demonstrated a good quality in terms of surface morphology, availability of numerous functional groups and amorphous nature of the adsorbent which could make it a potential candidate to tackle pollution challenges. The Rumex abyssinicus stem-based biosorbent has resulted in the 98.5 % maximum removal efficiency of methyl orange. This made the adsorbent effective at a low cost. On the other hand, the adsorption process accurately followed the Freundlich isotherm model, inferring heterogeneous and multilayer surface interaction. Additionally, the kinetics studies revealed that the data plot was inclined to a pseudo-second-order model and thermodynamics study analysis showed the nature of the adsorption to be feasible with spontaneous and endothermic adsorption processes. Finally, the low-cost, eco-friendly and locally available biosorbent derived from the stem of Rumex abyssinicus could be taken as an effective biosorbent for the decolorization of methyl orange-saturated industrial effluents.

## Compliance with ethical standards

Not applicable.

## Funding

No funding was received for this work.

## Data availability

Additional data is included in the supplementary material.

## Ethical clearance and consent to participate

Not applicable.

## Consent to publish

Nor applicable.

## CRediT authorship contribution statement

**Mikiyas Abewaa:** Writing – review & editing, Writing – original draft, Visualization, Software, Methodology, Investigation, Formal analysis, Data curation, Conceptualization. **Eba Adino:** Writing – review & editing, Writing – original draft, Conceptualization. **Ashagrie Mengistu:** Writing – review & editing, Writing – original draft.

## Declaration of competing interest

The authors declare that they have no known competing financial interests or personal relationships that could have appeared to influence the work reported in this paper.
