# Supplementary material for: Preparation of Rumex abyssinicus based biosorbent for the removal of methyl orange from aqueous solution
Source: Heliyon. 2023 Nov 20;9(12):e22447. doi: 10.1016/j.heliyon.2023.e22447 (PMC10716519; doi:10.1016/j.heliyon.2023.e22447)
Supplement: Multimedia component 1 [file mmc1.docx]

Table1. Removal efficiency of methyl orange adsorption

| Run No. | pH | Dye con.  (mg/L) | Adsorbent dosage (g/100ml) | Contact time (min) | Removal efficiency (%) | Absorbance |
| --- | --- | --- | --- | --- | --- | --- |
| 1 | 3 | 20 | 0.3 | 60 | 89.65 | 0.164511 |
| 2 | 9 | 10 | 0.3 | 60 | 59.24 | 0.179155 |
| 3 | 3 | 10 | 0.1 | 60 | 81.87 | 0.162635 |
| 4 | 3 | 20 | 0.2 | 60 | 73.43 | 0.188192 |
| 5 | 6 | 30 | 0.3 | 60 | 68.81 | 0.217706 |
| 6 | 6 | 20 | 0.1 | 60 | 82.95 | 0.174293 |
| 7 | 3 | 10 | 0.1 | 60 | 76.34 | 0.166672 |
| 8 | 9 | 10 | 0.2 | 60 | 72.86 | 0.169212 |
| 9 | 6 | 20 | 0.3 | 60 | 63.47 | 0.202734 |
| 10 | 9 | 20 | 0.1 | 60 | 55.54 | 0.214312 |
| 11 | 3 | 20 | 0.2 | 30 | 91.40 | 0.161956 |
| 12 | 3 | 20 | 0.3 | 30 | 94.26 | 0.15778 |
| 13 | 6 | 20 | 0.2 | 30 | 93.79 | 0.158467 |
| 14 | 3 | 30 | 0.2 | 30 | 84.38 | 0.183608 |
| 15 | 9 | 30 | 0.1 | 30 | 24.61 | 0.314504 |
| 16 | 6 | 10 | 0.1 | 30 | 97.06 | 0.151546 |
| 17 | 6 | 20 | 0.2 | 30 | 95.06 | 0.156612 |
| 18 | 6 | 30 | 0.3 | 30 | 90.24 | 0.170774 |
| 19 | 3 | 20 | 0.1 | 30 | 83.62 | 0.173315 |
| 20 | 6 | 30 | 0.2 | 30 | 87.45 | 0.176885 |
| 21 | 9 | 10 | 0.3 | 90 | 65.85 | 0.17433 |
| 22 | 3 | 20 | 0.1 | 90 | 78.68 | 0.180527 |
| 23 | 9 | 30 | 0.3 | 90 | 85.48 | 0.181199 |
| 24 | 9 | 10 | 0.2 | 90 | 90.21 | 0.156547 |
| 25 | 9 | 30 | 0.1 | 90 | 63.24 | 0.229904 |
| 26 | 9 | 10 | 0.1 | 90 | 81.14 | 0.163168 |
| 27 | 9 | 30 | 0.3 | 90 | 88.64 | 0.174278 |
| 28 | 6 | 30 | 0.3 | 90 | 86.47 | 0.179031 |
| 29 | 3 | 10 | 0.1 | 90 | 93.19 | 0.154371 |
| 30 | 6 | 30 | 0.2 | 90 | 79.08 | 0.195215 |

Table 2. Effect of contact time analysis on methyl orange removal efficiency

| Contact time | Removal efficiency | Absorbance |
| --- | --- | --- |
| 30 | 25.2 | 0.258608 |
| 45 | 75.4 | 0.185316 |
| 60 | 98.6 | 0.151444 |
| 75 | 84.8 | 0.171592 |
| 90 | 81.6 | 0.176264 |

Table 3. Effect of pH on methyl orange removal efficiency

| pH | Removal efficiency (%) | Absorbance |
| --- | --- | --- |
| 3 | 60.2 | 0.207508 |
| 4 | 78.8 | 0.180352 |
| 5 | 85.1 | 0.171154 |
| 6 | 98.4 | 0.151736 |
| 7 | 93.7 | 0.158598 |
| 8 | 76.6 | 0.183564 |
| 9 | 72.5 | 0.18955 |

Table 4. Effect of adsorbent dosage on methyl orange removal efficiency

| Adsorbent dosage (g/100 mL) | Removal efficiency (%) | Absorbance |
| --- | --- | --- |
| 0.1 | 68.1 | 0.195974 |
| 0.15 | 74.3 | 0.186922 |
| 0.20 | 98.4 | 0.151736 |
| 0.25 | 94.5 | 0.15743 |
| 0.30 | 90.6 | 0.163124 |

Table 5. Effect of initial dye concentration on methyl orange adsorption

| Initial dye concentration (mg/L) | Removal efficiency (%) | Absorbance |
| --- | --- | --- |
| 10 | 10 | 0.184075 |
| 15 | 15 | 0.193419 |
| 20 | 20 | 0.151298 |
| 25 | 25 | 0.170205 |
| 30 | 30 | 0.184002 |
